# Comparison of electroanatomic voltage mapping with late gadolinium enhancement CMR

**DOI:** 10.1186/1532-429X-16-S1-P153

**Published:** 2014-01-16

**Authors:** Dana C Peters, Warren J Manning, Mark E Josephson, James S Duncan, Sudhakar Chelikani

**Affiliations:** 1 Diagnostic Radiology, Yale School of Medicine, New Haven, Connecticut, USA; 2 Cardiovascular Medicine, Beth Israel Deaconess Medical Center, Boston, Massachusetts, USA; 3 Radiology, Beth Israel Deaconess Medical Center, Boston, Massachusetts, USA; 4 Biomedical Engineering, Yale University, New Haven, Connecticut, USA

## Background

Atrial fibrillation (AF) is associated with atrial remodeling, including atrial cellular pathology [1,2], left atrial enlargement [3], and low voltage on electroanatomic mapping (EAM)[4]. Recent studies have identified late gadolinium enhancement (LGE) in AF subjects pre-PVI as novel evidence of LA remodeling [5]. One study compared voltage by EAM obtained prior to a pulmonary vein isolation (PVI) procedure with the extent of atrial fibrosis using LGE [6]. Our goal was to further correlate EAM voltages with LGE.

## Methods

Sixteen AF patients were imaged on a 1.5T scanner (Achieva, Philips Healthcare, NL) using high resolution LGE [7], prior to their first PVI. Left atrial LGE was obtained using an ECG-triggered, navigator-gated 3D GRE inversion recovery (IR) sequence obtained 10-20 minutes after the administration of 0.2 mmol/kg of Gd-DTPA, with spatial resolution of 1.3 × 1.3 × 4 mm 3. The left atrial cavity and enhanced tissue were segmented on the LGE images, using a threshold corresponding to the signal of enhanced mitral valves. EAM was performed by CARTO (Biosense, Webster, Diamond Bar, CA, USA). The LGE enhancement and EAM data were registered using pulmonary vein ostia as landmarks, and fused. The mean bipolar voltage in regions with LA LGE enhancement vs. no LGE enhancement was calculated. Regions with no voltage mapping data (within 5 mm radius) were excluded. The areas of the LA cavity with low voltage (<0.38 mV) or with LGE were measured.

## Results

**Figure 1:**
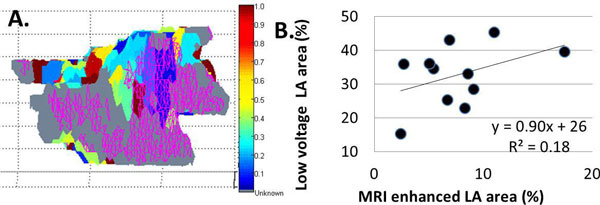
**A) Reconstructed bipolar voltage map merged with LGE enhancement (purple mesh)**. See voltage scale bar. B) Linear relationship between low voltage extent and LGE enhancement extent (p = 0.20).

Among all subjects, the mean bipolar voltage recorded in regions with LGE enhancement was 0.29 ± 0.17 mV vs. 0.51 ± 0.25 mV in regions without (p < 0.001). Figure 1A shows a representative map of LGE enhancement located in a region of low voltage. Figure 1B plots extent of low voltage vs. extent of LGE. Patients with more or less extensive LGE (using a cutoff of 6% by area) had similar BMI, LA volumes, hypertension, but older age correlated with greater LGE (Table 1).

**Table 1:** 

	All (N = 16)	More extensive LGE by area (%)	Less extensive LGE by area (%)
Age (years)*	58 ± 11	63 ± 7	51 ± 12

Male (%)	83%	62%	100%

Low voltage area (%)	33 ± 9%	35 ± 9%	29 ± 9%

BMI (kg/m^2)	26 ± 4	26 ± 4	28 ± 3

LA volume index	64 ± 20	62 ± 17	69 ± 23

Recurrence	63%	25%	50%

HTN	37%	37%	37%

*p = 0.03.

## Conclusions

Comparison of EAM and LGE enhancement/scar shows a relationship by which the voltage measured in enhanced regions of the atrial wall have lower average voltage, and a potential correspondence between LGE and EAM.

## Funding

Funding: NIH (NHLBI R21 HL 098573 & R21 HL103463).
